# Chronic asthma and Mesenchymal stem cells: Hyaluronan and airway remodeling

**DOI:** 10.1186/s12950-017-0165-4

**Published:** 2017-08-30

**Authors:** Benjamin D. Goldstein, Mark E. Lauer, Arnold I. Caplan, Tracey L. Bonfield

**Affiliations:** 1 Department of Pediatric Pulmonology, Rainbow Babies and Children’s Hospital, University Hospitals Cleveland Medical Center, Cleveland, OH USA; 2 Cleveland Clinic Foundation, Department of Biomedical Engineering, Cleveland, OH USA; 3 Skeletal Research Center, Department of Biology, Case Western Reserve University, Cleveland, OH USA; 4 Department of Pediatrics, Case Western Reserve University, Cleveland, OH USA; 5 Department of Pediatrics, Division of Pulmonary, Allergy and Sleep Medicine, 10900 Euclid Avenue, Biomedical Research Building #822, Cleveland, OH 44106-4948 USA

**Keywords:** Mesenchymal stem cells, Hyaluronan, Collagen, Extracellular matrix, Inflammation

## Abstract

**Background:**

Previous studies have demonstrated that ovalbumin sensitization promotes chronic asthma phenotype in murine asthma model. Human mesenchymal stem cells (hMSCs) are multipotent cells in vitro that have been shown to decrease inflammation and can reverse airway remodeling when infused into an in vivo chronic asthma model. However, the mechanism by which hMSCs reverse remodeling is still unclear. In this study, we hypothesized that hMSCs influence remodeling by decreasing extracellular matrix (ECM) deposition, more specifically by decreasing collagen I, collagen III, and hyaluronan synthesis.

**Methods:**

Chronic asthma phenotype was produced in an in vitro model with 3 T3 murine airway fibroblast cells by stimulating with GM-CSF. Collagen I and collagen III gene expression was investigated using RT-PCR and Taqman techniques. Hyaluronan was evaluated using FACE and Western Blots. The chronic asthma phenotype was produced in vivo in murine model using sensitization with ovalbumin with and without hMSC infusion therapy. ECM deposition (specifically trichrome staining, soluble and insoluble collagen deposition, and hyaluronan production) was evaluated. Image quantification was used to monitor trichrome staining changes.

**Results:**

GM-CSF which induced collagen I and collagen III production was down-regulated with hMSC using co-culture. In the in vivo model, Ovalbumin induced enhanced ECM deposition, soluble and insoluble collagen production, and lung elastance. hMSC infusions decreased ECM deposition as evidenced by decreases in soluble and insoluble collagen production.

**Conclusion:**

hMSCs participate in improved outcomes of remodeling by reversing excess collagen deposition and changing hyaluronan levels.

## Background

Asthma is the most common chronic disease in childhood. According to the Center for Disease Control(CDC), nearly one in ten children are affected [1]. The pathophysiology behind asthma is characterized by a Th2 inflammatory response which amounts to reversible airway hyper-responsiveness, inflammation, hypersecretion of mucus, and airway remodeling, all of which result in dynamic airway obstruction. During acute asthma exacerbations, which are the main causes of morbidity and mortality, there is an increase in symptoms usually due to known allergic (atopic) and non-allergic triggers. These exacerbations are usually the effect of viral illnesses commonly seen in the late fall to early winter months. Acute asthma treatment tends to be treated fairly quickly with the use of short-acting beta agonists (SABA) as well as short bursts of oral corticosteroids, frequently 3–5 days in duration. Unfortunately, these medications do not address the long-term control of asthma, or prevent hospitalizations. There are many available effective treatments to control baseline symptoms, including inhaled corticosteroids and leukotriene receptor antagonists [1–3]. The utilization of these therapeutics, however, attack the underlying inflammation but cannot reverse the chronic airway remodeling that results from the continued inflammatory insult to the lungs.

Airway remodeling, as seen in chronic asthma, includes epithelial detachment, subepithelial fibrosis, increased smooth muscle mass, goblet cell hyperplasia, proliferation of blood vessels, and edema. Subepithelial fibrosis was first described by Roche and colleagues in 1989 where bronchial biopsies were taken in patients with asthma. Based on histology and immunohistochemistry, it has been shown that there can be a dense collagen network underneath the true basement membrane, including fibronectin, proteoglycans, and collagen types I and III [2, 4]. The stimulus for airway remodeling is based on Th2 inflammation and chemotaxis of eosinophils. More specifically, eosinophils are produced in response to GM-CSF, IL4, and IL-13 and they produce pro-fibrotic mediators, especially TGF-beta [5–9].

Fibroblasts are mesenchymal cells that are found in most tissues including lungs which produce extracellular matrix proteins and collagen that give tissues shape and structure. Normally, these fibroblasts support controlled wound healing in areas of epithelial damage. However, in the process of airway remodeling in asthma, inflammation and exposures to environmental triggers can cause excessive epithelial damage and subsequent uncontrolled fibroblast migration, which results in subepithelial fibrosis [10, 11]. One of the important mediators in this process is IL-13 which stimulates fibroblast migration and invasion in asthma [12].

Hyaluronan (HA) is also a major component of the ECM. Hyaluronan is a non-sulfated glycosaminoglycan (repeating disaccharide) polymer that is synthesized by multiple cell types including stromal cells, fibroblasts, epithelial cells, and smooth muscle cells [13]. In the healthy lung, hyaluronan exists as high molecular mass (HMM HA), and is found within the peribronchial and perialveolar spaces. Within the ECM, HMM HA assists in cell movement, cushioning, and structural integrity [13]. HMM HA is produced by three different isoforms of hyaluronic acid synthase (HAS1, HAS2, and HAS3) with HAS2 as the major isoform in human lung fibroblasts and is consistently elevated in fibroblasts that have been differentiated into myofibroblasts in asthmatic airways [14, 15]. In the same study, it is of note that the asthmatic fibroblasts also increased collagen I and collagen III production.

Mesenchymal stem cells (MSCs) are a group of multipotent, self-renewing progenitor cells that can be derived from multiple tissue types, including bone marrow [16], umbilical cord [17], and compact bone [18]. MSCs have been shown to differentiate in vitro into many different cell phenotypes including cells which make bone, cartilage, muscle, bone marrow, tendon/ligament, adipose tissue, and other connective tissue types [16, 17, 19]. Previously, it has been shown that MSCs can produce IL-1RA, which blocks macrophage activation through IL-1 alpha and IL1-beta [20–22], and TGF-beta, which suppresses macrophage and T cell activation [23–25]. The ability of MSCs to produce significant amounts of cytokines and proteins is important in that these MSCs can alter the immune system by decreasing inflammation and promote wound healing. As such, MSC-based therapy represents a potential cure for asthma and is the focus of our study.

We hypothesized that fibroblasts modulate the amount of airway subepithelial fibrosis by producing collagen I, collagen III, and hyaluronan synthase 2. MSC therapy results in decreased expression of collagen and hyaluronan synthase 2 which would ultimately decrease subepithelial fibrosis and degenerative airway remodeling in chronic asthma.

## Methods

### Cell models

#### Cell sources

NIH 3 T3 murine fibroblast cells (ATCC CRL-1658) were grown in specialized DMEM high glucose media (Gibco, cat #11885–092) containing 10% heat-inactivated FCS (Life Technologies, cat #16010–159), and 1% Penicillin-Streptomycin-Glutamine (PSG) (Gibco, cat #10378–016). Mesenchymal stem cells (MSCs) were received in collaboration with The Skeletal Research Center at Case Western Reserve University in Cleveland, OH and were grown as previously described elsewhere [11]. Briefly, the MSCs were grown in DMEM containing 10% fetal bovine serum from highly selected sources [26]. Within 14 days, the colony-forming units that were selectively expanded to near confluence were lifted from the culture dish using trypsin and re-plated at 1:3 to insure that they maintain active cell division.

#### Cell harvest

cells were be mechanically removed from culture plates using plunger from 1 mL syringe, centrifuged at 1800 rpm for 9 min, and supernatant and cell pellet will be stored separately at −80 °C as previously described by Jainchill and colleagues [27].

#### Co-culture of 3 T3 fibroblasts and hMSC supernatants

NIH 3 T3 murine fibroblast cells were grown as previously described above. When 3 T3 fibroblast cells were 80% confluent, cells with and without stimulation had their growth medium changed to medium obtained from hMSCs.

### Gene expression studies

#### RNA isolation & cDNA synthesis

3 T3 murine fibroblasts were lysed with RiboZol reagent (Fisher Scientific, cat #50–751-7365) and washed with chloroform (Fisher Scientific, cat # BP 1145-1 L). The lysed cells were centrifuged at 12,000 g for 15 min at 5 °C to achieve phase separation, and the RNA-containing aqueous layer was removed. The RNA was precipitated using isopropanol, centrifuged at 12,000 g for 10 min at 5 °C, washed three times with 70% ethanol, centrifuged at 7500 g for 5 min at 5 °C, and dissolved in sterile nuclease-free water (Fisher Scientific, cat # BP2484–100) at 55 °C. The cDNA was synthesized using the qScript cDNA kit (VWR, cat #101414–098) and stored at −20 °C or used immediately for Real Time-PCR (RT-PCR). RNA and cDNA concentration will be determined by spectrophotometry (NanoDrop ND-1000, Thermo Fisher). cDNA was diluted in sterile nuclease-free water to which Taqman Universal PCR Master Mix (Life Technologies, cat #4304437) and PCR primers (Life Technologies) were added. Samples were plated in a 96-well reaction plate (Applied BioSystems, cat #4306737). The reaction was run on an Applied BioSystems 7300 Real Time PCR System and analyzed using ABI 7300 Prism software and Microsoft Excel. Data was normalized to mouse GAPDH.

### Mouse model

All procedures involving mice were reviewed and approved by the Case Western Reserve University Institutional Animal Care and Use Committee. BALB/c mice were purchased from The Jackson Laboratories (Bar Harbor, ME) and sensitized by intra-peritoneal injections (100uL) of ovalbumin emulsified in 1.5 mg of Al(OH)3 as previously described [11]. On day 14, mice were exposed to 1% wt/vol ovalbumin in PBS by intranasal challenge every other day for 8 weeks. Sham sensitization and challenges were carried out with sterile PBS. During the last week of the challenge (week 8), mice were given 1 × 106 MSCs via retro-orbital administration. For each study, there were 7–10 animals in each group (ovalbumin-sensitized saline challenge without or without hMSCs, ovalbumin-sensitized ovalbumin challenge with and without hMSCs), which were subsequently split into processing for pathology (2–3 mice, see description below).

### Lung pathology

Animals for each of the groups above were euthanized without BAL to preserve the intact lung architecture. Following perfusion with paraformaldehyde, the lungs were embedded in paraffin and sectioned at 4 μm and mounted for evaluation trichrome staining. Sections were controlled for proximal airways for comparison purposes. For each of the groups, quantitative measurements of inflammation were made using Image-Pro Plus software (Media Cybernetics, Bethesda, MD) as previously described [28] which is an automated program which allows from more than 196 different sections to be analyzed off the same tissue section on a given slide. Each data point represents an *n* = 3 different experiment with 4 mice per group.

### Soluble and insoluble collagen assays

#### Hydroxyproline assay (insoluble assay)

The snap-frozen lungs were thawed and washed twice in sterile PBS with 2 complete tablets of proteinase/protease inhibitor (Life Sciences, cat # 04693116001). The lungs were placed in plastic tubes with 700uL CHAPS buffer (50 mM Tris-HCl buffer, pH 7.4 + 150 mM NaCl +10 mM CHAPS) and 700uL PBS, and homogenized for 2 min. The supernatant was split into three 1.5 mL Eppendorf tubes (one for hydroxyproline assay, one for Sirius Red Assay, and one for RT-PCR assay). The hydroxyproline tube was centrifuged in speed vacuum for 45 min until all liquid is evaporated. Following vacuum spin, 2 mL of 6 N HCl was added to each tube and covered; place in dry bath at 110 °C for 24 h. The following day, the glass tubes were returned to the speed vacuum for 3 h, then allowed to cool at room temperature. Once cooled, 300uL of citrate/acetate buffer (5 g citric acid +1.2 mL glacial acetic acid +7.24 g sodium acetate +3.4 g NaOH) was placed in each sample tube, and filtered into another 1.5 mL Eppendorf tube, and 1 mL Chloramine T solution (0.282 g Chloramine T + 2 mL n-propanol +2 mL distilled water +16 mL citrate/acetate buffer) was added. This mixture was incubated at room temperature for 20 min. Following this incubation period, 1 mL Ehrlich’s solution (4.6 g 4-(dimethyalamino)-benzaldehyde +18.6 mL n-propanol +7.8 mL 70% perchloric acid) was added to each sample and incubated for 20 min at 65 °C. The sample were allowed to cool for 10 min at room temperature after which 200uL of standard stock solutions (hydroxyproline and citrate/acetate buffer) were added to the 96-well plate with 200uL of each sample evaluated in a spectrophotometer at 540 nm.

#### Sirius red tissue staining (soluble assay)

One-third of whole lung homogenate was placed in 1.5 mL Eppendorf tube. The tube was centrifuged at 10,000 g for 10 min. Subsequently, the resultant supernatant was placed in 100uL aliquots. Ten microliters of sample were removed and plated into 96-well plate with 140uL PBS. The plate was dried overnight at 37 °C. The following day, the plate was washed with 200uL/well of distilled water three times, after which 150uL of Sirius Red Stain (Sigma Aldrich, cat # 365548-25G) was added to each well. The plate was incubated at room temperature on a rocker for 1 h after which the plate was washed again four times with 200 uL/well of acidified water (5% acetic acid). After washing with acidified water, 150uL of NaOH (0.1 M NaOH) was added to each well and incubated at room temperate for 30 min on a rocker. After transferring to a new 96-well plate, the samples were evaluated in a spectrophotometer at 550 nm. Insoluble and soluble collagen was be measured by colorimetric assays (QuickZyme Biosciences).

### Hyaluronan analysis

#### Fluorophore-assisted carbohydrate electrophoresis (FACE)

To determine the state and quantity of HA, lung homogenates were processed using FACE [29]. The process involves isolating and breaking down the HA into its component disaccharides which are fluorescently labeled and then processed with gel electrophoresis and digital imaging.

#### Agarose gel electrophoresis (HSAE)

Differentiating low molecular weight vs. high molecular weight HA was determined using HA analysis by agarose gel electrophoresis or HSAE. This technique is used for qualitative analysis of the size of the HA chains using proteases and nucleases to remove proteins and nucleic acid from the same leaving behind the glycosaminoglycans (GAGs) which are subsequently separated by size on agarose gel and then stained for HA. The HA size was determined by comparing to a ladder of purified HA with known molecular weight. Low molecular weight HA is <500 kDa, and high molecular weight HA is >500 kDa. Additionally, the lung homogenates were evaluated for HA synthase gene expression analyzing mRNA expression using Taqman technique as described above.

### Statistical analysis

The quantitative outcomes were evaluated by group comparisons with respect to measurements at individual time points. They comparisons were described numerically by means, standard deviations, and appropriate percentiles. For group and time point comparisons, we used repeated measures analysis of variance (ANOVA) and t-tests. If there was a lack of normality, then non-parametric Wilcoxon rank sum test and Friedman’s tests was considered. A *p* value of 0.05 was considered significant for all tests.

## Results

### Cell lines studies

**Fig. 1:**
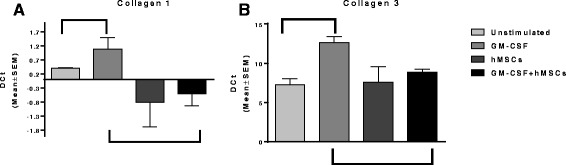
3 T3 fibroblasts were grown in the presence and absence of GM-CSF and MSCs). GM-CSF increased the gene expression of collagen type I, III and HAs relative to the expression of these genes in 3 T3 cells at baseline without stimulation (*p* ≤ 0.05, *n* = 3). Using this modeling system of increased production of ECM related molecules we tested the capacity of bone marrow derived hMSC supernatants to alter the up-regulated ECM genes with GM-CSF. Treatment of the GM-CSF treated 3 T3 cells with hMSC supernatants decreased collagen type I, (1**a**) III (**1b**) and HAs gene expression (*p* ≤ .05, *n* = 3)

To assess the impact of hMSCs on lung remodeling, we utilized an in vitro model using 3 T3 murine fibroblast cells. Using these cells, we developed an assay which demonstrated the effectiveness of hMSCs on collagen production, specifically looking at collagen I and collagen III mRNA synthesis expression in 3 T3 murine fibroblast cells stimulated with GM-CSF. In these studies we also evaluated the expression of hyaluronan synthesase (HAs). HAs is the enzyme which is required for the generation of hyaluronan and contributor to inflammation and remodeling. We measured mRNA gene expression using Taqman technology as described in Methods section; the results can be seen in Fig. 1.

In Fig. 1a and b, 3 T3 fibroblasts were grown in the presence and absence of GM-CSF and MSCs. GM-CSF significantly increased collagen I, collagen III and HA synthetase (HAs) mRNA expression (*p* < 0.05, *n* = 3). Both the collagen I and collagen III, gene expression levels were subsequently reversed in the presence of hMSCs supernatants (*p* < 0.05, *n* = 3). The corresponding b﻿ars within each figure represent statistical significant differences between groups. These results showed that in this setting of inflammation induced by GM-CSF, collagen I, III, mimicking in vitro extracellular matrix changes due to inflammation supernatants from hMSCs have the capacity to reverse the synthesis of collagen genes. The impact of the hMSC superanatant on the 3 T3 expression of HAs did result in decreased mRNA synthesis, but only by 8 ± 3.5%, which was just out of the range of statistical significance (*p* = 0.06, *n* = 3). In these studies, treatment with hMSCs had the capacity to transcriptionally decrease the gene expression of both collagen type I and type III as well as the synthesis of HAs the enzyme responsible for the production of HA.

### Mouse model

#### Extracellular matrix deposition and remodeling

**Fig. 2:**
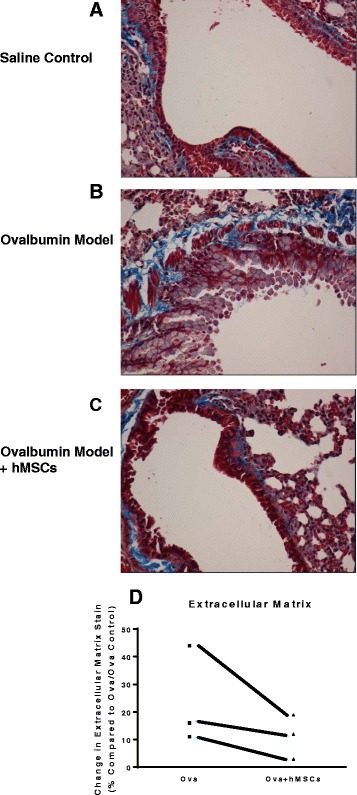
MSCs and ECM. Using the in vivo model of chronic asthma and ovalbumin as the antigen, mice were evaluated for the amount of ECM by trichrome staining (*bright blue color*). Compared to controls (**a**), the animals that were challenged with ovalbumin had higher levels of trichrome bright blue staining (**b**). In each study group, an subset of mice were given 10^6^ hMSCs. Treatment with hMSCs cells decreased the lung distribution of trichrome staining (**c**) consistent with the decrease in ECM. ImagePro quantitative analysis was used to quantify the amount of bright blue ECM defined by the trichrome staining in 198 sectional images of each tissue section in each study of a different hMSC donor. For each experimental series, in which there were controls (sensitized but not challenged), asthma (sensitized and challenged) and treated group (sensitized, challenged and treated with hMSCs) there were at least 10 animals in a group. Each group experimental series used a different hMSC donor. (**d**) The distribution of ECM was significantly decreased within each experimental series between the OVA and OVA treated with hMSCs(*p* = 0.04, *n* = 3 experiments including each group, statistics used regression mutli-variant analsyis)

Using the ovalbumin model of asthma, the impact on hMSCs on ECM generated in response to allergic challenge was monitored by the change in trichrome staining. Trichrome stains the collagen components, reflected in bright blue staining in Fig. 2. The amount of trichrome staining was quantified by our ImagePro Program [28]. Since the impact of the stem cell therapy could be very site specific, imaging was done on both left and right lung lobes, slicing through the large and small airways (*n* = 5, animals per experiment, 3 different experiments). Visually, an example of the model and enhanced ECM with challenge and the quantification are shown in Fig. 2. The OVA challenge for 8 weeks enhanced ECM production relative to the saline control (Fig. 2a vs. b) as evidenced by the increased amount of bright blue staining, documented by the ImagePro analysis. To evaluate the impact of hMSC on ECM as measured anatomically, mice were treated with 10^6^ hMSC through the retro-orbital sinus as described previoiusly [11]. Visually the amount of Trichrome staining decreased in the hMSC treated groups (Fig. 2c , *n* = 5 animals per experiment, 3 different experiments). Using the same ImagePro program, several sections of the lung tissue, were evaluated for changes in ECM deposition. In each of the three different experiments, the administration of hMSCs decreased quantitative levels of ECM from the amount of ECM that would be present in the sham treated- OVA challenged animals (Fig. 2c).

#### Insoluble and soluble collagen

**Fig. 3:**
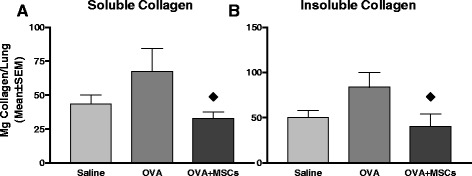
Soluble and Insoluble Collagen Changes with MSCs. Whole lung homogenates were obtained from the 4-week chronic asthma model (obtained from the same three studies used for the imaging analysis). Lungs were processed for either soluble (sirius red assay) or insoluble (hydroxyproline Assay). In **a** and **b**, the diamond represents a decrease in collagen deposition in response to hMSCs. Each bar is 7 animals representative of a single chronic asthma study (*p* = 0.06, F variance *p* = 0.05)

To determine the effect on structural components of the lungs we chose to evaluate the amount of ECM deposition in our chronic asthma model using a monitor of total soluble and insoluble collagen in mice sensitived and challenged with OVA. Soluble and insoluble collagen measues in complex in vivo tissues can be a good monitor of therapeutic impact. Insoluble collagen is associated with increased tissue collagen in the structural component of the lungs. The soluble collagen levels are associated with initiation of repair against remodeling. The animal model was evaluated with or without the infusion of hMSCs. The insoluble and soluble collagen was measured by hydroxyproline and Sirius Red colorimetric assays, respectively. In Fig. 3a, OVA sensitized mice had increased amount of soluble collagen when measured using Sirius Red tissue staining and this collagen deposition was decreased in mice injected with hMSCs. In Fig. 3b, insoluble collagen deposition was measured using hydroxyproline assays. The amount of insoluble collagen in the lungs of OVA sensitized mice was increased when compared to controls, and this deposition was decreased in mice infused with hMSCs. These studies demonstrate hMSC infusion decreased insoluble and soluble ECM deposition in the form of collagen.

#### Hyaluronan analysis

**Fig. 4:**
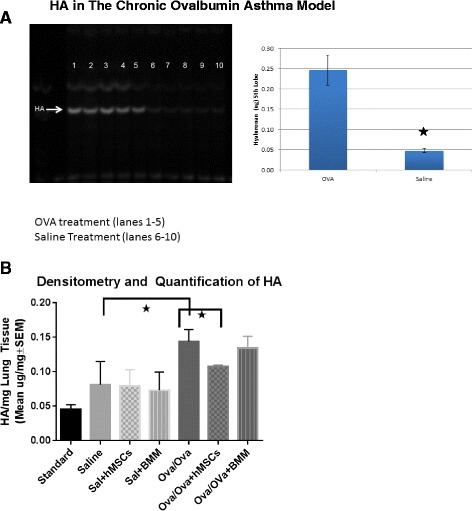
Whole lung homogenates were obtained from the asthma model with and without MSCs or BMMs and evaluated for HA. Ovalbumin challenge mice had elevated HA (4**a**, *p* ≤ 0.05, *n* = 5). Treatment of the mice with 10^6^ hMSCs resulted in decreased levels of HA (4**b**, *p* ≤ 0.05, *n* = 5). Treatment by the control cells, in these studies bone marrow cells BMM, had no impact on the amount of challenge induced HA *vertical lined bar*. The saline group which received hMSCs had no change in HA levels. T﻿he bars in figure 4b represent statistical significant (Ova/Ova) differences in hyaluronan production. The first bar represents an increase in hyaluronan in OVA sensitized and OVA challenged mice vs. saline shame (light solid bar). The second bar represents a significant decrease in hyaluronan production in OVA sensitized/challenged mice who received hMSCs (cross-hatched bar)

Previous studies have established that elevated hyaluronan (HA) in the lung is associated with inflammation in asthma [30]. There are several methods to monitor shifts in hyaluronan in tissue. FACE technology is useful in complex tissues since it allows for semi-quantification of total HA, as well as the strunctural identiy of the HA regarding the change in TSG-6 mediated glycosylation and HA converting from low molecular weight to high molecular forms which are pathogenic [29, 30]. HSAE technology, however, is able to qualitatively detect differences in size to distinguish high molecular weight vs. low molecular weight hyaluronan. As previously demonstrated in the literature [30], OVA sensitization and challenge statistically increased HA in the lungs (Fig. 4a, *n* = 3, *p* < 0.05). hMSCs infusion significantly decreased HA (Fig. 4b, *n* = 3, *p* < 0.05).

## Discussion

Chronic asthma is characterized by chronic inflammation and airway remodeling, which includes infiltration of inflammatory cells and abnormal accumulation of extracellular matrix (ECM). There are multiple components of the ECM with airway fibroblasts as the main cell type that producing this ECM them. Multiple studies have shown that in chronic asthma, there are increases in collagen synthesis and deposition as well hyaluronan deposition. The current treatment modalities for chronic asthma include inhaled corticosteroids, combination therapy of inhaled corticosteroids and long acting beta-adrenergic agonists, oral leukotriene receptor antagonists, and chronic oral steroids. MSCs offer another possible treatment modality that could possibly reverse or prevent chronic inflammation seen in chronic asthma and possibly offer improved side effect profiles than the current treatment regimens do not offered.

In our study, we use both in vitro and in vivo models. In the in vitro model, 3 T3 murine fibroblasts had increased levels of col. 1, col. 3 and HAs mRNA synthesis in response to GM-CSF stimulation as a model of increased airway remodeling and fibrosis (collagen expression) in response to inflammation and GM-CSF a relevant stimulus in asthma. In the in vivo model, OVA sensitized and challenged mice had increased ECM deposition as evidenced by increased trichrome staining. We were also able to show that OVA sensitized and challenged mice had increased soluble and insoluble collagen production which was decreased by hMSC infusion. Hyaluronan (HA) was increased in OVA sensitized and challenged mice and this was decreased by hMSC infusion. This study suggests a possible mechanism by which hMSCs decreased airway remodeling and fibrosis as evidenced by our in vitro and in vivo measurements *of* collagen production and deposition, ECM deposition, and HA production. Soluble and insoluble measures of collagen were not utilized in the in vitro 3 T3 assay since, the point of the outcome measure was to evaluate changes in complex tissues comprising more than just fibroblastic cells, which would not be representative of the soluble and insoluble measure.

One of the limitations in our study was the use of OVA protein which has not been shown to be pathogenic in human models. There are current studies, however, investigating sensitization with house dust mite antigens (Der P1 among others) and its ability to induce chronic inflammation and, subsequently, whether MSCs can reduce and possibly inhibit chronic inflammation [31, 32]. The house dust mite antigen-sensitized murine model is likely to be more translational to human models.

In a recent study by Yilmaz et al. provided a review of remodeling and inflammation in pediatric asthma as well as possible therapeutic agents [33]. The authors discussed remodeling of the airways and provide pathophysiologic mechanisms such as epithelial/subepithelial tissue rearrangement, sub-epithelial fibrosis, epithelial basement membrane thickening, myofibroblast hyperplasia, increased smooth muscle layer thickness, and neoangiogenesis [33]. Chronic inflammation lead to excess fibroblast response which increased ECM deposition resulting in remodeling [34, 35]. Our study provides additional insight into the underlying mechanism and also document that hMSCs might provide provided another therapeutic modality.

In conclusion, our study offers an insight into the possible mechanism of hMSCs therapy inhibiting chronic inflammation seen in a chronic asthma model induced by OVA sensitization. Future studies are needed to elucidate these mechanisms and to see whether these mechanisms are consistent in the house dust mite antigen model.

## Conclusion

Chronic asthma is characterized by reversible airway hyper-responsiveness, inflammation, hypersecretion of mucus, and airway remodeling. Airway remodeling, as seen in chronic asthma, includes epithelial detachment, sub-epithelial fibrosis, increased smooth muscle mass, goblet cell hyperplasia, proliferation of blood vessels, and edema. The pathophysiology behind airway remodeling, especially sub-epithelial fibrosis, includes increased extracellular matrix deposition. This ECM deposition is made up of multiple types of components such as collagen I, collagen III, and the glycosaminoglycan hyaluronan. We were able to show a possible mechanism for hMSCs decreasing chronic inflammation in a cell-based and murine asthma model.

## Abbreviations

ECM: Extracellular matrix
FACE: Fluorophore-assisted carbohydrate electrophoresis
GAG: Glycosaminoglycan
GM-CSF: Granulocyte macrophage colony stimulating factor
HA: Hyaluronan/hyaluronic acid
HAS: Hyaluronan synthase
HSAE: Agarose gel electrophoresis
IL: Interleukin
MSC: Mesenchymal stem cell
OVA: Ovalbumin
SABA: Short acting beta agonist
TGF-beta: Transforming growth factor beta
